# Combining Biomarkers to Predict Pregnancy Complications and Redefine Preeclampsia: The Angiogenic-Placental Syndrome

**DOI:** 10.1161/HYPERTENSIONAHA.119.13763

**Published:** 2020-02-17

**Authors:** Holger Stepan, Martin Hund, Theresa Andraczek

**Affiliations:** 1 From the Department of Obstetrics, Leipzig University, Leipzig, Germany (H.S., T.A.); 2 Roche Diagnostics International, Ltd, Rotkreuz, Switzerland (M.H.).

## Abstract

Placental dysfunction underlies a spectrum of perinatal pathologies, including preeclampsia and fetal growth restriction. Angiogenesis-related factors, including sFlt-1 (soluble fms-like tyrosine kinase 1) and PlGF (placental growth factor), play an important role in placental dysfunction; altered levels are detectable several weeks before onset of pregnancy complications. In vitro diagnostic tests for these biomarkers can improve early diagnosis and facilitate prediction of maternal and fetal outcomes. We assessed evidence for combining angiogenic biomarkers with other biomarkers or clinical parameters to predict maternal/fetal outcomes in pregnant women with placental dysfunction. Pooled information on placental perfusion (ultrasonography, mean arterial pressure), clinical characteristics, and biomarker levels (PlGF) can improve first-trimester prediction and preeclampsia diagnosis. Angiogenic factors (sFlt-1/PlGF ratio; PlGF alone) with or without clinical characteristics can facilitate second-/third-trimester prediction of early-onset and late-onset preeclampsia. A combination of increased sFlt-1/PlGF ratio and ultrasound can rule out early fetal growth restriction. The sFlt-1/PlGF ratio is also a reliable tool for discriminating between pregnancy-related hypertensive disorders, including superimposed preeclampsia and gestational hypertension. Analysis of angiogenic factors with or without uterine Doppler substantially improves sensitivity and specificity for predicting adverse outcomes and iatrogenic preterm delivery. We propose to extend the American College of Obstetricians and Gynecologists definition of preeclampsia in the future to include the combination of new-onset hypertension and new-onset of altered angiogenic factors (sFlt-1/PlGF ratio or PlGF alone). In summary, altered angiogenic biomarkers indicate placental dysfunction, and their implementation into clinical practice will help reduce the considerable burden of morbidity and mortality associated with adverse pregnancy outcomes as a consequence of angiogenic-placental syndrome.

## Abstract 2

Supplemental Digital Content is available in the text.

Placental dysfunction (PD), originally described in 1948, underlies a spectrum of obstetric and perinatal pathologies, including preeclampsia, fetal growth restriction (FGR), and placental abruption.^1,2^ The pathophysiology of PD is conventionally characterized as involving a defective deep trophoblastic invasion and impaired maternal spiral artery remodeling, leading to inadequate placental perfusion during the second half of pregnancy.^3^ However, this understanding has been challenged by recent findings indicating that most histological changes observed in PD are unspecific.^4^ In particular, the established concept and phenomenon of a shallow trophoblast invasion is a more typical feature of pregnancies with early-onset preeclampsia or FGR. Evidence from a systematic review of early-onset preeclampsia studies showed that the majority of preeclampsia cases had a normal placenta and that villous lesions were also present in a proportion of normal pregnancies.^5^ Maternal factors such as microvillous overcrowding may contribute to the development of late-onset preeclampsia as placental growth reaches its limits.^6^

Several angiogenic factors play an important role in PD: VEGF-A (vascular endothelial growth factor A) is essential for placental vascular development, affecting proliferation and migration of endothelial cells and vascular permeability; PlGF (placental growth factor), a proangiogenic VEGF family member, is abundantly expressed in the placenta and acts by enhancing the action of VEGF-A; sFlt-1 (soluble fms-like tyrosine kinase 1), an antiangiogenic VEGF family member, is important in the regulation of angiogenic homeostasis during pregnancy. sFlt-1 and PlGF are expressed in the placenta and extra-placentally, in vascular endothelial cells, fibroblasts, osteoblasts, smooth muscle cells, and monocytes.^4^ An imbalance between pro and antiangiogenic factors (ie, increased sFlt-1/PlGF ratio) results in a net antiangiogenic state and favors the development of PD (Figure 1).^6-8^ sFlt-1, PlGF, and sEng (soluble endoglin) are important biomarkers for PD.^9^

**Figure 1.**
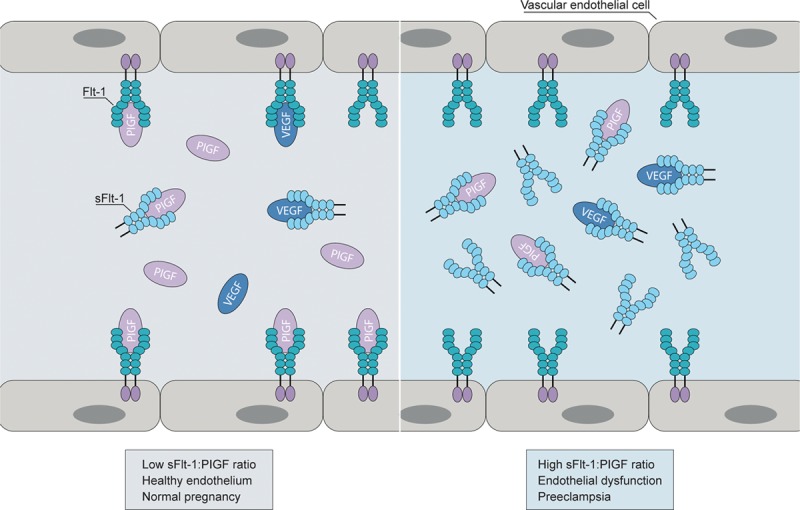
Role of sFlt1 (soluble fms-like tyrosine kinase 1) and PlGF (placental growth factor) in preeclampsia development. In pregnant women, sFlt-1 binds and inhibits VEGF (vascular endothelial growth factor) and PlGF in the circulation. A high sFlt-1/PlGF ratio, therefore, potentially results in endothelial dysfunction and development of preeclampsia. Reprinted from Benzing^8^ with permission. Copyright ©2016, Springer Nature.

Diagnostic criteria for PD are currently based on nonspecific clinical, ultrasound, and laboratory findings and offer little predictive capability for adverse fetal and maternal outcomes.^10^ As altered levels of angiogenic factors are detectable weeks before onset of pregnancy complications, in vitro diagnostic tests for these biomarkers can improve early diagnosis and facilitate prediction of maternal and fetal outcomes. We examine how combining angiogenic biomarkers with other biomarkers/clinical parameters at different time points can predict maternal/fetal outcomes in pregnant women with PD.

## Preeclampsia and Hemolysis, Elevated Liver Enzymes, and Low Platelet Count Syndrome

### Overview and Diagnosis

**Figure 2.**
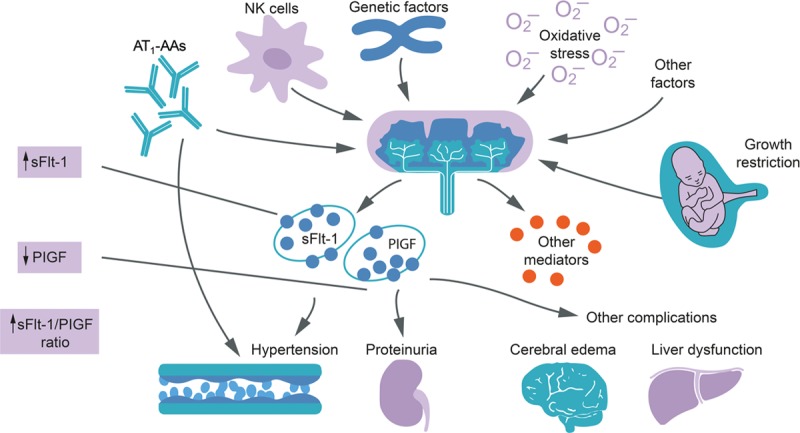
Pathophysiology and features of preeclampsia. Altered angiogenic factors indicating placental dysfunction can result in diverse adverse pregnancy outcomes. AT1-AAs indicates agonistic angiotensin II type 1 receptor autoantibodies; NK, natural killer; PlGF, placental growth factor; and sFlt-1, soluble fms-like tyrosine kinase 1. Reprinted from Wang et al^12^ with permission. Copyright ©2009, American Physiological Society.

Preeclampsia is a hypertensive syndrome affecting 2% to 3% of pregnancies and characterized by endothelial damage in multiple organs (Figure 2).^10–12^ The definition of preeclampsia previously included hypertension plus proteinuria after 20 weeks’ gestation and has expanded to include hypertension in combination with renal and liver dysfunction and thrombocytopenia (Table 1).^10,11,13^ Proteinuria is no longer necessary to fulfill the definition of preeclampsia. Preeclampsia can be classified into early-onset (<34 weeks’ gestation) and late-onset (≥34 weeks’ gestation).^14^ Disease severity varies from mild through to severe, culminating in potentially life-threatening end-stage complications, such as hemolysis, elevated liver enzymes, and low platelet count syndrome, and eclamptic seizures.^14,15^

**Table 1.**
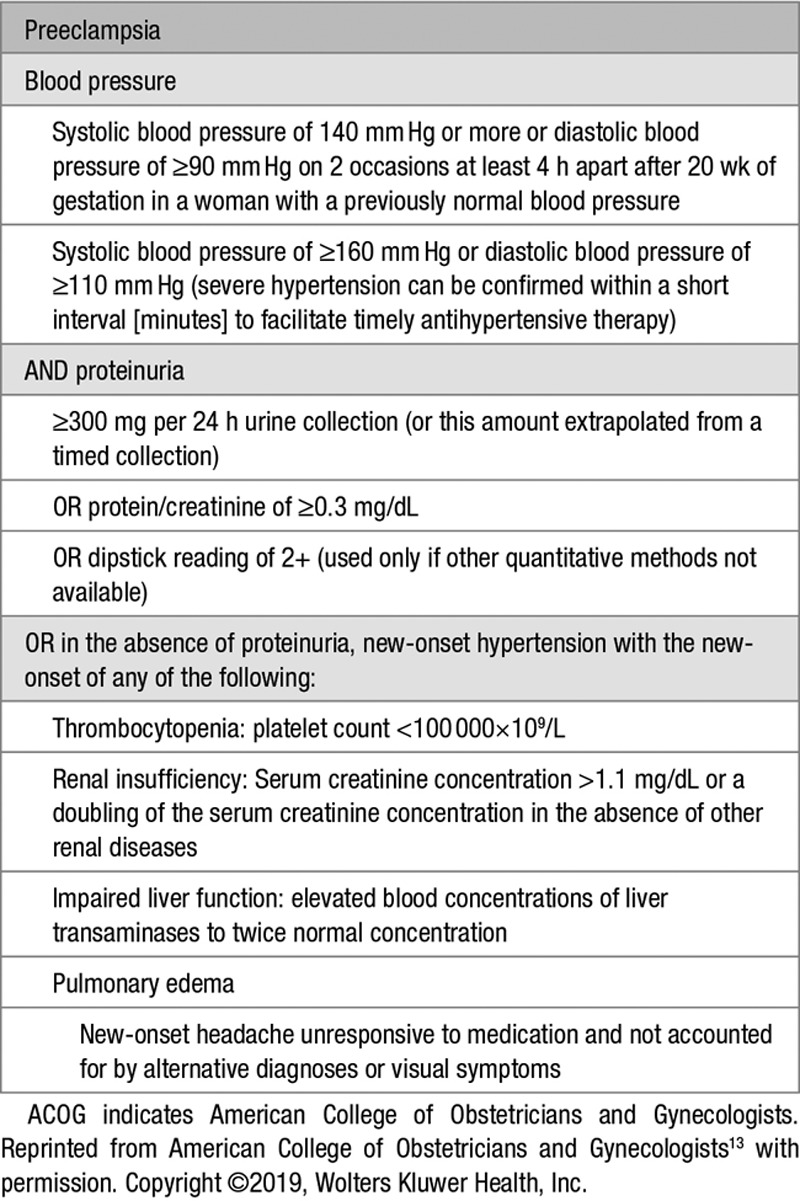
Clinical Definition of Preeclampsia (ACOG Criteria)^10,13^

The current gold standard for preeclampsia diagnosis relies on observation of new-onset hypertension and proteinuria during the second half of pregnancy and has poor predictive ability for preeclampsia-related adverse outcomes.^10^ Some evidence-based guidelines currently include use of angiogenic biomarkers in the context of preeclampsia. The National Institute for Health and Care Excellence recommends use of the Elecsys sFlt-1/PlGF ratio alongside standard clinical assessment to help rule out preeclampsia in women presenting with suspected preeclampsia between 20 weeks and 34 weeks plus 6 days of gestation.^16^ The guidelines of the German Society of Obstetrics and Gynecology, Austrian Society of Obstetrics and Gynecology, and Swiss Society of Obstetrics and Gynecology on hypertensive disorders in pregnancy recommend the use of angiogenic biomarkers to aid diagnosis and short-term prediction of preeclampsia in pregnant women with suspected disease.^17^ The use of the sFlt-1/PlGF ratio for ruling out preeclampsia in pregnant women with suspected preeclampsia is recommended in the 2018 European Society of Cardiology guidelines for the management of cardiovascular diseases during pregnancy.^18^

Although biomarkers have been shown to predict and diagnose preeclampsia,^19^ recent evidence supports the use of combinations of biomarkers with or without other clinical measurements to better determine the clinical problem and outcome.

### First-Trimester Preeclampsia Prediction

Use of the Fetal Medicine Foundation algorithm can improve first-trimester prediction and diagnosis of preeclampsia. The Fetal Medicine Foundation algorithm combines information on risk factors, such as placental perfusion (uterine artery pulsatility index [UtA-PI] plus mean arterial pressure), clinical characteristics (maternal factors/medical history), and biomarker levels (PlGF), to estimate risk for preeclampsia. Considerable evidence supports this combined approach,^20–24^ including 3 large-scale prospective cohort studies in women who attended their routine first hospital visit at 11 to 13 weeks’ gestation. In the first study, combined screening at a false-positive rate (FPR) of 10% predicted 75% of preterm preeclampsia and 47% of term preeclampsia and was superior to screening based on maternal factors alone.^20^ The combined approach is also superior to guideline-based methods.^21,23^ O’Gorman et al^21^ reported superior performance of the combined approach for predicting preeclampsia (10% FPR: <32 weeks, 100% detection rate [DR]; <37 weeks, 75% DR; ≥37 weeks, 43% DR) compared with methods recommended by National Institute for Health and Care Excellence (10.2% FPR: <32 weeks, 41% DR; <37 weeks, 39% DR; ≥37 weeks, 34% DR) and the American College of Obstetricians and Gynecologists (ACOG; 64.2% FPR: <32 weeks, 94% DR; <37 weeks, 90% DR; ≥37 weeks, 89% DR). These findings were confirmed in the Screening program for pre-eclampsia (SPREE) study, where the combined approach provided a DR for preterm preeclampsia of 82.4% versus 40.8% using the National Institute for Health and Care Excellence method.^22^ Pooled analysis of these 3 studies (61 174 women with singleton pregnancies; 1770 cases of preeclampsia) demonstrated that combined screening detected 90% of early preeclampsia (<32 weeks), 75% of preterm preeclampsia (<37 weeks), and 41% of term preeclampsia (≥37 weeks), at a 10% FPR.^23^ Therefore, the predictive value of the combined approach is generally greater for early-onset preeclampsia than for late-onset preeclampsia.

### Second- or Third-Trimester Preeclampsia Prediction

Angiogenic factors (eg, sFlt-1/PlGF ratio or PlGF alone) with or without clinical characteristics information can facilitate second- or third-trimester prediction of early- and late-onset preeclampsia.^25–30^

The pivotal PRediction of short-term Outcome in preGNant wOmen with Suspected preeclampsIa Study (PROGNOSIS) study evaluated the sFlt-1/PlGF ratio for predicting absence or presence of preeclampsia in 1050 women with suspected preeclampsia (24+0 to 36+6 weeks’ gestation). This prospective, multicenter study derived and validated an sFlt-1/PlGF ratio of ≤38 for ruling out preeclampsia within 1 week—in the validation arm, a cutoff of ≤38 provided a negative predictive value (NPV) of 99.3% (Table 2).^25^ The clinical utility of the sFlt-1/PlGF ratio cutoff of 38 for short-term prediction of preeclampsia was validated in 764 Asian pregnant women with suspected preeclampsia in PROGNOSIS Asia. In this study, the sFlt-1/PlGF ratio of ≤38 had an NPV of 98.6% (95% CI, 97.2%–99.4%) for ruling out preeclampsia within 1 week.^31^ An sFlt-1/PlGF ratio ≤38 at 36 weeks’ gestation was also clinically useful for ruling out severe preeclampsia among low-risk patients in an unselected cohort of nulliparous women (NPV, 99.2%).^27^ Sabrià et al^28^ showed that between 24 and 34 weeks’ gestation, no subsequent determination was needed to completely rule out early-onset preeclampsia when the first sFlt-1/PlGF ratio determination was ≤38, in singleton pregnancies with signs or symptoms of this syndrome. A single third-trimester sFlt-1/PlGF ratio measurement can predict late-onset preeclampsia and FGR with a sensitivity and specificity of 84.4% and 93.0%, respectively.^32^ A recent meta-analysis and systematic review also concluded that the sFlt-1/PlGF ratio can prove a valuable screening tool for preeclampsia, helping in decision-making, treatment stratification, and better resource allocation.^29^

**Table 2.**
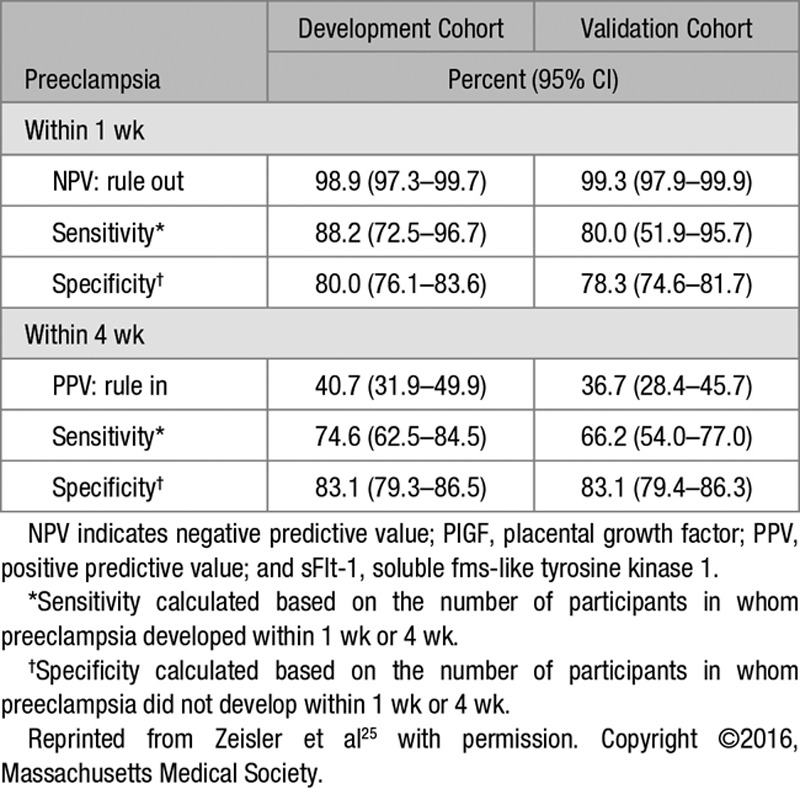
Validation of a sFlt-1/PlGF Ratio Cutoff of ≤38 for Predicting Preeclampsia^25^

Recently, a prediction model for early-onset preeclampsia was developed, which included the sFlt-1/PlGF ratio plus mean arterial pressure, being parous, and previous preeclampsia; this model was superior to those using the sFlt-1/PlGF ratio alone or with mean UtA-PI.^26^ Consistent with these findings, Gómez-Arriaga et al^33^ showed the sFlt-1/PlGF ratio is superior to Doppler for predicting preeclampsia in women with singleton pregnancies and suspected or confirmed preeclampsia. In early preeclampsia, mean UtA-PI at diagnosis was abnormal in 100% and 91% of cases with and without FGR; sFlt-1/PlGF was abnormal in 100% and 96% of cases, respectively. In contrast, in late preeclampsia, mean UtA-PI was abnormal in 50% and 37% of cases with and without FGR, whereas the sFlt-1/PlGF ratio was abnormal in 50% and 26% of cases, respectively. The authors concluded that mean UtA-PI was not diagnostically useful in late preeclampsia and that the sFlt-1/PlGF ratio showed high specificity but low sensitivity to confirm suspected late-onset preeclampsia.

It should be noted that other angiogenic factors can also provide predictive value for preeclampsia. For example, in pregnant women with abnormal uterine perfusion, combined analysis of second-trimester sEng and sFlt-1 predicted early-onset preeclampsia with a sensitivity of 100% and a specificity of 93.3%.^30^

## Fetal Growth Restriction

### Overview and Diagnosis

FGR describes reduced fetal growth velocity whereby the fetus fails to achieve its full growth potential. Early FGR (<32 weeks’ gestation) accounts for 20% to 30% of cases and is associated with underlying placental pathology in addition to preeclampsia, whereas late FGR (≥32 weeks’ gestation) accounts for ≈ 70% of cases and is less strongly associated with hypertensive disorders.^34,35^ Although there is no gold standard definition, a widely used proxy is delivery of a small-for-gestational-age (SGA) infant (10th percentile) and an adverse pregnancy outcome. Clinically, SGA is characterized by a small fetus and normal uterine/umbilical Doppler, whereas FGR is characterized by a small fetus and an abnormal Doppler. Biomarkers, which are altered in FGR and usually normal in SGA, offer an additional means to differentiate placentally mediated and constitutionally small fetuses. However, there is a considerable overlap between early preeclampsia and FGR, as highlighted in the Trial of randomized umbilical and fetal flow in Europe (TRUFFLE) study, which evaluated management strategies for early FGR.^36^ In this multicenter randomized trial, the majority of pregnancies had to be delivered preterm due to maternal complications, such as hypertensive diseases, including preeclampsia.^36^

### Biomarkers and Ultrasound for FGR Prediction

Combining low PlGF or increased sFlt-1/PlGF ratio (indicating PD) and ultrasound (detecting SGA fetus <5th or 10th percentile) can diagnose FGR.^35,37–41^ Benton et al^37^ showed that in women with suspected FGR (ultrasound <10th percentile for gestational age), PlGF alone (<5th percentile by gestational age) had 98.2% sensitivity, 75.1% specificity, 99.2% NPV, and 58.5% positive predictive value (PPV) for identifying pregnancies with underlying placental pathology. Addition of sFlt-1 information, that is, sFlt-1/PlGF ratio, can provide additional predictive value. Gaccioli et al^38^ reported an association between a combination of elevated sFlt-1/PlGF ratio (>85th percentile) and ultrasonically suspected SGA at 28 and 36 weeks’ gestational age (<10th percentile at 28 weeks; sFlt-1/PlGF cutoff of 38 at 36 weeks) in a prospective cohort of 4512 nulliparous women. The diagnostic effectiveness of this approach at 28 weeks’ gestation for preterm delivery of an SGA infant was characterized by 38.5% sensitivity, 99.1% specificity, 21.3% PPV, and 99.6% NPV. Similarly, diagnostic effectiveness at 36 weeks’ gestation for preterm delivery of an SGA infant associated with maternal preeclampsia or perinatal morbidity/mortality was characterized by 37.9% sensitivity, 97.8% specificity, 21.6% PPV, and 99.0% NPV (Table S1 in the online-only Data Supplement).^38^ Consistent with these findings, MacDonald et al^40^ demonstrated clinical utility of sFlt-1, PlGF, and their ratio for detecting SGA infants or preeclampsia at 36 weeks’ gestation. Median plasma concentrations of PlGF were significantly lower in women who subsequently had SGA new-borns (178.5 versus 326.7 pg/mL; *P*<0.0001), and the sensitivity and specificity of sFlt-1/PlGF ratio (cutoff 33.4) to predict <10th percentile SGA infants were 26.5% and 89.9%, respectively. For comparison, a strategy of selective third-trimester ultrasound provided 22.9% sensitivity for SGA.^40^

Finally, preliminary evidence from a prospective longitudinal study suggests a combination of sFlt-1/PlGF and NT-proBNP (N-terminal prohormone of brain natriuretic peptide) may predict isolated FGR; this approach is in the early stages of investigation and not used in clinical practice.^42^

These findings illustrate that combined information on angiogenic biomarkers and ultrasound generally provides higher specificity (rule out) than sensitivity (rule in) for FGR and that specificity for predicting adverse outcomes is increased by knowledge of the sFlt-1/PlGF ratio. Furthermore, early and late FGR appear to have distinct pathologies, with angiogenic biomarkers showing greater relevance in early FGR. Thus, the clinical use of angiogenic biomarkers to facilitate early FGR diagnosis is attractive, as early FGR is difficult to manage (in contrast to late FGR that is more difficult to diagnose but easier to manage).^43^

## Superimposed Preeclampsia, Chronic Hypertension, and Gestational Hypertension

### Overview and Diagnosis

Gestational hypertension is characterized by elevated blood pressure during the second half of pregnancy in previously normotensive women, whereas superimposed preeclampsia (SPE) is the development of preeclampsia in women with preexisting (ie, chronic) hypertension. Preeclampsia occurs 3 to 5× more frequently in women with preexisting hypertension compared with women who are normotensive at conception.^44^ However, diagnosis of SPE based on current recommendations is challenging, as women already have hypertension and may already exhibit proteinuria.

### sFlt-1/PlGF Ratio for SPE or Gestational Hypertension Prediction

The sFlt-1/PlGF ratio is a reliable tool for discriminating between pregnancy-related hypertensive disorders. Patients with preeclampsia or hemolysis, elevated liver enzymes, and low platelet count syndrome have a significantly increased sFlt-1/PlGF ratio compared with patients with normal pregnancy outcomes or chronic and gestational hypertension (*P*<0.001).^45^ A higher sFlt-1/PlGF ratio can facilitate diagnosis of early-onset SPE but is less predictive of late-onset SPE where angiogenic imbalance is less prominent.^46,47^ For example, in women with preexisting chronic hypertension, the sFlt-1/PlGF ratio is higher before clinical diagnosis at 20 weeks’ gestation in individuals who subsequently developed early-onset SPE between 28 and 34 weeks, compared with levels in those who never developed preeclampsia (*P*=0.001) or who developed late-onset SPE (*P*=0.001).^46^ Furthermore, addition of the sFlt-1/PlGF ratio to a model comprising information on systolic blood pressure, serum uric acid, and plasma renin activity can improve predictive accuracy.^46^

Despite a role in SPE, angiogenic imbalance appears to play a lesser role than in preeclampsia. Costa et al^47^ showed that individuals with preeclampsia had significantly higher sFlt-1/PlGF ratios than normotensive women at gestational weeks 26 (*P*=0.004), 32 (*P*=0.001), and 36 (*P*=0.029). In contrast, women with SPE only had a higher sFlt-1/PlGF ratio at week 32 (*P*=0.039), compared with women who remained chronically hypertensive.

Women with chronic kidney disease (CKD) frequently develop SPE, and distinction from underlying disease can be challenging.^48,49^ Bramham et al^49^ examined the diagnostic performance of PlGF, sFlt-1, and the sFlt-1/PlGF ratio for predicting SPE in women with and without CKD or chronic hypertension. Women with SPE and requiring delivery within 14 days had higher sFlt-1/PlGF ratios than women with CKD or chronic hypertension without SPE (*P*<0.0001). Diagnostic performance of the sFlt-1/PlGF ratio for SPE requiring delivery within 14 days in women with CKD or chronic hypertension was confirmed (receiver operator characteristic area under the curve, 0.83; SE, 0.06). Consistent with these findings, an observational study of pregnant women with CKD demonstrated a significant increase in sFlt-1 and significant decrease in PlGF in pregnancies with SPE, compared with women showing severe proteinuria without hypertension or a normal clinical course and normal controls.^50^

## Adverse Pregnancy Outcomes

### Overview

The overall burden of adverse pregnancy-related disease is considerable. Preterm and low birth weight are the most relevant biologic determinants of new-born infant survival, with preterm births accounting for 75% of perinatal mortality and >50% of long-term morbidity.^51^ Stillbirths also represent a substantial global burden with an estimated average worldwide rate of 18.4 per 1000 births in 2015.^52^ Placental abruption affects 0.4% to 1.0% of pregnancies and is itself associated with increased risk of preterm birth, stillbirth, and perinatal mortality, the latter extending beyond the perinatal period.^53^ Several recent studies show a clear association between adverse pregnancy outcomes and an imbalance in angiogenic regulators.^54–56^

### sFlt-1, PlGF, and Ultrasound for Adverse Pregnancy Outcomes Prediction

**Figure 3.**
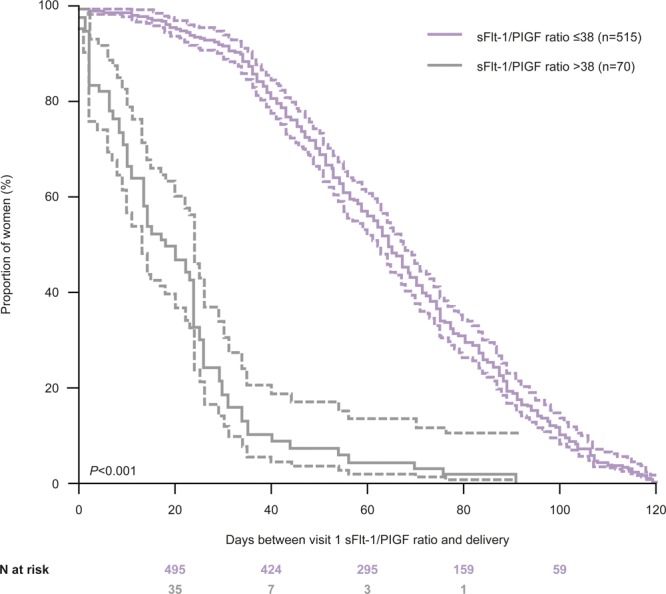
Kaplan-Meier curve of time to delivery according to sFlt-1-to-PlGF (soluble fms-like tyrosine kinase 1 to placental growth factor) ratio at visit 1 in women without preeclampsia (samples taken during early gestational phase). Reprinted from Zeisler et al^12^ with permission. Copyright ©2016, The American College of Obstetricians and Gynecologists.

Adverse outcomes are here defined as unintended events occurring as a result of medical care and are harmful to a patient’s health. In the context of PD, the most common adverse outcome is iatrogenic preterm delivery. Combined analysis of uterine Doppler and angiogenic factors substantially improves sensitivity and specificity for prediction of adverse outcomes and iatrogenic preterm delivery.^42,56–61^ Stepan et al^58^ observed significantly higher sFlt-1 (1403.6±555 versus 451.8±42 pg/mL; *P*<0.05) and lower PlGF (139.6±24 versus 184.1±21 pg/mL) concentrations in second-trimester pregnancies with adverse versus normal outcomes—this difference was more pronounced in patients with subsequent preeclampsia than subsequent FGR due to greater decrease in PlGF in preeclampsia. Second-trimester assessment of sFlt-1 and Doppler also improved sensitivity for iatrogenic preterm delivery before 34 weeks’ gestation. Sensitivity and specificity, respectively, for prediction of preterm delivery, were 64% and 63% with Doppler alone, 79% and 78% with sFlt-1+PlGF, 71% and 76% with sFlt-1/PlGF ratio, and 79% and 80% with Doppler plus sFlt-1.^58^ Similarly, in women diagnosed with FGR before 34 weeks’ gestation via ultrasonography, an sFlt-1/PlGF cutoff value of ≥86.2 predicted adverse outcomes with a 77.8% sensitivity and 80.0% specificity. Notably, a high sFlt-1/PlGF ratio was also associated with a shorter duration to delivery (*P*<0.001; Figure 3).^57,62^

### Biomarkers Alone for Adverse Pregnancy Outcomes Prediction

In the absence of clinical measurements, combined biomarker and single biomarker measurements can facilitate prediction of adverse pregnancy outcomes. Patients with an intermediate sFlt-1/PlGF ratio (>38 and <85) are at risk for severe adverse outcomes^63,64^ and those with sFlt-1/PlGF ratio >38 have a shorter remaining pregnancy duration and higher risk of preterm delivery.^31,62^ In a study of women with suspected preeclampsia (24–36 6/7 weeks’ gestation), individuals with sFlt-1/PlGF ratio >38 had a 2.9-fold greater likelihood of imminent delivery (*P*<0.001) and shorter remaining time to delivery (median 17 versus 51 days; *P*<0.001) compared with those with sFlt-1/PlGF ratio ≤38, regardless of the development of preeclampsia.^62^ Similarly, in the PROGNOSIS Asia study, individuals with a sFlt-1/PlGF ratio >38 had a greater risk of imminent delivery (hazard ratio, 3.5) compared with individuals with a ratio ≤38.^31^ Preliminary investigations also suggest that NT-proBNP can predict preterm delivery and may increase the predictive accuracy of an algorithm based on sFlt-1/PlGF plus gestational age at measurement.^42,65^

Finally, a recent systematic review identified PlGF as a predictor of adverse intrapartum and perinatal outcomes.^66^ Low PlGF levels were consistently associated with cesarean section for fetal compromise, neonatal intensive care unit admission, and stillbirth.

## Refining the Definition of Preeclampsia: the Angiogenic-Placental Syndrome

Preeclampsia was previously defined as hypertension plus proteinuria after 20 weeks’ gestation. However, the term preeclampsia only describes a symptom before eclampsia and is rather unspecific. The designation of preeclampsia based on the old definition, including hypertension and proteinuria, is now outdated, as hypertension and proteinuria are only 2 among many other symptoms with poor predictive value. The growing understanding of preeclampsia as a heterogeneous hypertensive disorder of pregnancy triggered the ACOG’s hypertension 2013 task force to revise the definition of preeclampsia to include the presence of severe features with or without proteinuria (Table 1).^10,13^ Clinical and pathological studies suggest that the placenta plays a central role in the pathogenesis of preeclampsia.^67^ Altered angiogenic biomarkers (sFlt-1/PlGF ratio or PlGF alone) are indicative of PD. sFlt-1/PlGF ratio of ≥85 is associated with the diagnosis of preeclampsia and predicted adverse outcomes and delivery within 2 weeks.^16,68^ Low PlGF in pregnant women indicates PD with its clinical correlate of preeclampsia or fetal growth restriction.^69^

Therefore, in the interest of fostering discussion around this important area, it is our opinion that the ACOG definition of preeclampsia could be extended to include the combination of new-onset hypertension and new-onset altered angiogenic biomarkers (Tables 1 and 3). This approach would be consistent with the updated 2019 guideline of the German, Swiss and Austrian Societies of Obstetrics and Gynecology for hypertensive disorders in pregnancy, where combined new-onset hypertension plus altered angiogenic status (increased sFlt-1/PlGF ratio or decreased PlGF alone) not accounted for by alternative diagnoses is recognized as preeclampsia.^17^ Moreover, we suggest that the term preeclampsia should be evolved to angiogenic-placental syndrome. Angiogenic factors, in combination with new-onset hypertension as an aid to diagnose preeclampsia, show high specificity (low FPR), in particular for early-onset preeclampsia (99.5% specificity shown for sFlt-1/PlGF ratio using a cutoff of 85).^70,71^ Adding the angiogenic factor sFlt-1 to PlGF alone (yielding the sFlt-1/PlGF ratio) increases specificity for diagnosis of preeclampsia (reduced FPR).^72^ To minimize the FPR in late-onset preeclampsia the cutoff values for altered angiogenic factors may be adjusted (such as higher cutoff value of sFlt-1/PlGF ratio) in combination with new-onset hypertension.^71^ This could be implemented in a diagnostic algorithm reflecting the ACOG definition of preeclampsia.

Moreover, this approach would support physicians and aid identification of pregnant women with SPE and pregnant women developing preeclampsia in whom onset of proteinuria is observed much later than the onset of hypertension. The designation angiogenic-placental syndrome better reflects the pathobiology of distinct clinical manifestations resulting from a placenta-generated antiangiogenic state.

**Table 3.**
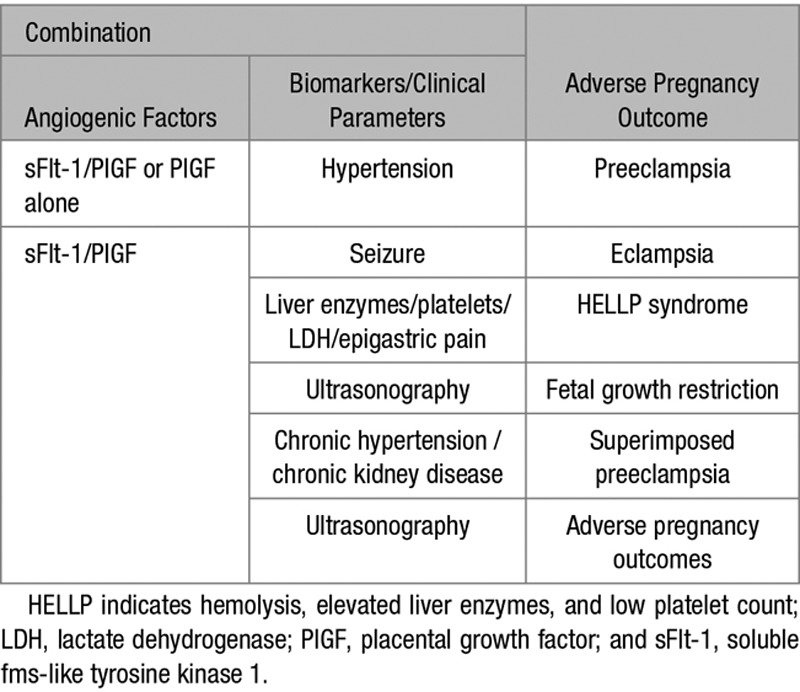
Concept of Combining Angiogenic Factors With Other Biomarkers/Clinical Parameters to Predict Adverse Pregnancy Outcomes

## Sources of Funding

Medical writing support was provided by Chloe Fletcher (Gardiner Caldwell Communications) and funded by Roche Diagnostics International, Ltd.

## Disclosures

H. Stepan received consultant and speaker fees from Roche Diagnostics, Thermo Fisher, and Kaneka. M. Hund is an employee of Roche Diagnostics International, Ltd, and holds stock in Hoffmann-La Roche. T. Andraczek reports no conflicts. ELECSYS is a trademark of Roche.

## Supplementary Material



## References

[1] Herraiz I, Simón E, Gómez-Arriaga PI, Martínez-Moratalla JM, García-Burguillo A, Jiménez EA, Galindo A (2015). Angiogenesis-Related biomarkers (sFlt-1/PLGF) in the prediction and diagnosis of placental dysfunction: an approach for clinical integration.. Int J Mol Sci.

[2] Page EW (1948). Placental dysfunction in eclamptogenic toxemias.. Obstet Gynecol Surv.

[3] Roberts JM (2014). Pathophysiology of ischemic placental disease.. Semin Perinatol.

[4] Huppertz B (2020). Biology of preeclampsia: combined actions of angiogenic factors, their receptors and placental proteins.. Biochim Biophys Acta Mol Basis Dis.

[5] Falco ML, Sivanathan J, Laoreti A, Thilaganathan B, Khalil A (2017). Placental histopathology associated with pre-eclampsia: systematic review and meta-analysis.. Ultrasound Obstet Gynecol.

[6] Redman CW, Sargent IL, Staff AC (2014). IFPA senior award lecture: making sense of pre-eclampsia - two placental causes of preeclampsia?. Placenta.

[7] Maynard SE, Min JY, Merchan J, Lim KH, Li J, Mondal S, Libermann TA, Morgan JP, Sellke FW, Stillman IE (2003). Excess placental soluble fms-like tyrosine kinase 1 (sFlt1) may contribute to endothelial dysfunction, hypertension, and proteinuria in preeclampsia.. J Clin Invest.

[8] Benzing T (2016). Hypertension: Testing for pre-eclampsia: paving the way to early diagnosis.. Nat Rev Nephrol.

[9] Levine RJ, Lam C, Qian C, Yu KF, Maynard SE, Sachs BP, Sibai BM, Epstein FH, Romero R, Thadhani R, CPEP Study Group (2006). Soluble endoglin and other circulating antiangiogenic factors in preeclampsia.. N Engl J Med.

[10] American College of Obstetricians and Gynecologists (2013). Hypertension in pregnancy: executive summary.. Obstet Gynecol.

[11] Tranquilli AL, Dekker G, Magee L, Roberts J, Sibai BM, Steyn W, Zeeman GG, Brown MA (2014). The classification, diagnosis and management of the hypertensive disorders of pregnancy: a revised statement from the ISSHP.. Pregnancy Hypertens.

[12] Wang A, Rana S, Karumanchi SA (2009). Preeclampsia: the role of angiogenic factors in its pathogenesis.. Physiology (Bethesda).

[13] American College of Obstetricians and Gynecologists (2019). ACOG Practice Bulletin No. 202: gestational hypertension and preeclampsia.. Obstet Gynecol.

[14] Tranquilli AL, Brown MA, Zeeman GG, Dekker G, Sibai BM (2013). The definition of severe and early-onset preeclampsia. Statements from the International Society for the Study of Hypertension in Pregnancy (ISSHP).. Pregnancy Hypertens.

[15] Munro PT (2000). Management of eclampsia in the accident and emergency department.. J Accid Emerg Med.

[16] National Institute for Health and Care Excellence (NICE) (2016). PlGF-based testing to help diagnose suspected pre-eclampsia (Triage PlGF test, Elecsys immunoassay sFlt-1/PlGF ratio, DELFIA Xpress PlGF 1-2-3 test, and BRAHMS sFlt-1 Kryptor/BRAHMS PlGF plus Kryptor PE ratio).

[17] German Society of Obstetrics and Gynecology (DGGG), OEGG and SGGG. Guidelines for Hypertensive Disorders in Pregnancy Diagnosis and therapy.. https://www.awmf.org/leitlinien/detail/ll/015-018.html.

[18] Regitz-Zagrosek V, Roos-Hesselink JW, Bauersachs J, Blomström-Lundqvist C, Cífková R, De Bonis M, Iung B, Johnson MR, Kintscher U, Kranke P, ESC Scientific Document Group (2018). 2018 ESC Guidelines for the management of cardiovascular diseases during pregnancy.. Eur Heart J.

[19] Stepan H, Herraiz I, Schlembach D, Verlohren S, Brennecke S, Chantraine F, Klein E, Lapaire O, Llurba E, Ramoni A (2015). Implementation of the sFlt-1/PlGF ratio for prediction and diagnosis of pre-eclampsia in singleton pregnancy: implications for clinical practice.. Ultrasound Obstet Gynecol.

[20] O’Gorman N, Wright D, Casanova C, Campanero M, Nicolaides KH (2016). Competing risks model in screening for preeclampsia by maternal factors and biomarkers at 11-13 weeks gestation.. Am J Obstet Gynecol.

[21] O’Gorman N, Wright D, Poon LC, Rolnik DL, Syngelaki A, de Alvarado M, Carbone IF, Dutemeyer V, Fiolna M, Frick A (2017). Multicenter screening for pre-eclampsia by maternal factors and biomarkers at 11-13 weeks’ gestation: comparison with NICE guidelines and ACOG recommendations.. Ultrasound Obstet Gynecol.

[22] Tan MY, Wright D, Syngelaki A, Akolekar R, Cicero S, Janga D, Singh M, Greco E, Wright A, Maclagan K (2018). Comparison of diagnostic accuracy of early screening for pre-eclampsia by NICE guidelines and a method combining maternal factors and biomarkers: results of SPREE.. Ultrasound Obstet Gynecol.

[23] Tan MY, Syngelaki A, Poon LC, Rolnik DL, O’Gorman N, Delgado JL, Akolekar R, Konstantinidou L, Tsavdaridou M, Galeva S (2018). Screening for pre-eclampsia by maternal factors and biomarkers at 11-13 weeks’ gestation.. Ultrasound Obstet Gynecol.

[24] Bahlmann F, Al Naimi A (2016). Using the angiogenic factors sFlt-1 and PlGF with Doppler ultrasound of the uterine artery for confirming preeclampsia.. Arch Gynecol Obstet.

[25] Zeisler H, Llurba E, Chantraine F, Vatish M, Staff AC, Sennström M, Olovsson M, Brennecke SP, Stepan H, Allegranza D (2016). Predictive value of the sFlt-1:PlGF ratio in women with suspected preeclampsia.. N Engl J Med.

[26] Perales A, Delgado JL, de la Calle M, García-Hernández JA, Escudero AI, Campillos JM, Sarabia MD, Laíz B, Duque M, Navarro M, STEPS Investigators (2017). sFlt-1/PlGF for prediction of early-onset pre-eclampsia: STEPS (Study of Early Pre-eclampsia in Spain).. Ultrasound Obstet Gynecol.

[27] Sovio U, Gaccioli F, Cook E, Hund M, Charnock-Jones DS, Smith GC (2017). Prediction of preeclampsia using the soluble fms-Like tyrosine kinase 1 to placental growth factor ratio: a prospective Cohort Study of unselected nulliparous women.. Hypertension.

[28] Sabrià E, Lequerica-Fernández P, Ganuza PL, Ángeles EE, Escudero AI, Martínez-Morillo E, Alvárez FV (2018). Use of the sFlt-1/PlGF ratio to rule out preeclampsia requiring delivery in women with suspected disease. Is the evidence reproducible?. Clin Chem Lab Med.

[29] Agrawal S, Cerdeira AS, Redman C, Vatish M (2018). Meta-Analysis and systematic review to assess the role of soluble FMS-like tyrosine kinase-1 and placenta growth factor ratio in prediction of preeclampsia: The SaPPPhirE Study.. Hypertension.

[30] Stepan H, Geipel A, Schwarz F, Krämer T, Wessel N, Faber R (2008). Circulatory soluble endoglin and its predictive value for preeclampsia in second-trimester pregnancies with abnormal uterine perfusion.. Am J Obstet Gynecol.

[31] Bian X, Biswas A, Huang X, Lee KJ, Li TK, Masuyama H, Ohkuchi A, Park JS, Saito S, Tan KH (2019). Short-Term prediction of adverse outcomes using the sFlt-1 (Soluble fms-Like Tyrosine Kinase 1)/PlGF (Placental Growth Factor) ratio in Asian women with suspected preeclampsia.. Hypertension.

[32] Birdir C, Droste L, Fox L, Frank M, Fryze J, Enekwe A, Köninger A, Kimmig R, Schmidt B, Gellhaus A (2018). Predictive value of sFlt-1, PlGF, sFlt-1/PlGF ratio and PAPP-A for late-onset preeclampsia and IUGR between 32 and 37 weeks of pregnancy.. Pregnancy Hypertens.

[33] Gómez-Arriaga PI, Herraiz I, López-Jiménez EA, Gómez-Montes E, Denk B, Galindo A (2013). Uterine artery doppler and sFlt-1/PlGF ratio: usefulness in diagnosis of pre-eclampsia.. Ultrasound Obstet Gynecol.

[34] Audette MC, Kingdom JC (2018). Screening for fetal growth restriction and placental insufficiency.. Semin Fetal Neonatal Med.

[35] Crovetto F, Triunfo S, Crispi F, Rodriguez-Sureda V, Roma E, Dominguez C, Gratacos E, Figueras F (2016). First-trimester screening with specific algorithms for early- and late-onset fetal growth restriction.. Ultrasound Obstet Gynecol.

[36] Lees C, Marlow N, Arabin B, Bilardo CM, Brezinka C, Derks JB, Duvekot J, Frusca T, Diemert A, Ferrazzi E, TRUFFLE Group (2013). Perinatal morbidity and mortality in early-onset fetal growth restriction: cohort outcomes of the trial of randomized umbilical and fetal flow in Europe (TRUFFLE).. Ultrasound Obstet Gynecol.

[37] Benton SJ, McCowan LM, Heazell AE, Grynspan D, Hutcheon JA, Senger C, Burke O, Chan Y, Harding JE, Yockell-Lelièvre J (2016). Placental growth factor as a marker of fetal growth restriction caused by placental dysfunction.. Placenta.

[38] Gaccioli F, Sovio U, Cook E, Hund M, Charnock-Jones DS, Smith GCS (2018). Screening for fetal growth restriction using ultrasound and the sFLT1/PlGF ratio in nulliparous women: a prospective cohort study.. Lancet Child Adolesc Health.

[39] Kienast C, Moya W, Rodriguez O, Jijón A, Geipel A (2016). Predictive value of angiogenic factors, clinical risk factors and uterine artery doppler for pre-eclampsia and fetal growth restriction in second and third trimester pregnancies in an Ecuadorian population.. J Matern Fetal Neonatal Med.

[40] MacDonald TM, Tran C, Kaitu’u-Lino TJ, Brennecke SP, Hiscock RJ, Hui L, Dane KM, Middleton AL, Cannon P, Walker SP (2018). Assessing the sensitivity of placental growth factor and soluble fms-like tyrosine kinase 1 at 36 weeks’ gestation to predict small-for-gestational-age infants or late-onset preeclampsia: a prospective nested case-control study.. BMC Pregnancy Childbirth.

[41] Triunfo S, Crovetto F, Rodriguez-Sureda V, Scazzocchio E, Crispi F, Dominguez C, Gratacos E, Figueras F (2017). Changes in uterine artery doppler velocimetry and circulating angiogenic factors in the first half of pregnancies delivering a small-for-gestational-age neonate.. Ultrasound Obstet Gynecol.

[42] Sabriá E, Lequerica-Fernández P, Lafuente-Ganuza P, Eguia-Ángeles E, Escudero AI, Martínez-Morillo E, Barceló C, Álvarez FV (2018). Addition of N-terminal pro-B natriuretic peptide to soluble fms-like tyrosine kinase-1/placental growth factor ratio > 38 improves prediction of pre-eclampsia requiring delivery within 1 week: a longitudinal cohort study.. Ultrasound Obstet Gynecol.

[43] Caradeux J, Martinez-Portilla RJ, Peguero A, Sotiriadis A, Figueras F (2019). Diagnostic performance of third-trimester ultrasound for the prediction of late-onset fetal growth restriction: a systematic review and meta-analysis.. Am J Obstet Gynecol.

[44] Zetterström K, Lindeberg SN, Haglund B, Hanson U (2005). Maternal complications in women with chronic hypertension: a population-based cohort study.. Acta Obstet Gynecol Scand.

[45] Verlohren S, Herraiz I, Lapaire O, Schlembach D, Moertl M, Zeisler H, Calda P, Holzgreve W, Galindo A, Engels T (2012). The sFlt-1/PlGF ratio in different types of hypertensive pregnancy disorders and its prognostic potential in preeclamptic patients.. Am J Obstet Gynecol.

[46] Perni U, Sison C, Sharma V, Helseth G, Hawfield A, Suthanthiran M, August P (2012). Angiogenic factors in superimposed preeclampsia: a longitudinal study of women with chronic hypertension during pregnancy.. Hypertension.

[47] Costa RA, Hoshida MS, Alves EA, Zugaib M, Francisco RP (2016). Preeclampsia and superimposed preeclampsia: The same disease? The role of angiogenic biomarkers.. Hypertens Pregnancy.

[48] Williams D, Davison J (2008). Chronic kidney disease in pregnancy.. BMJ.

[49] Bramham K, Seed PT, Lightstone L, Nelson-Piercy C, Gill C, Webster P, Poston L, Chappell LC (2016). Diagnostic and predictive biomarkers for pre-eclampsia in patients with established hypertension and chronic kidney disease.. Kidney Int.

[50] Masuyama H, Nobumoto E, Okimoto N, Inoue S, Segawa T, Hiramatsu Y (2012). Superimposed preeclampsia in women with chronic kidney disease.. Gynecol Obstet Invest.

[51] Goldenberg RL, Culhane JF, Iams JD, Romero R (2008). Epidemiology and causes of preterm birth.. Lancet.

[52] Blencowe H, Cousens S, Jassir FB, Say L, Chou D, Mathers C, Hogan D, Shiekh S, Qureshi ZU, You D, Lawn JE, Lancet Stillbirth Epidemiology Investigator G (2016). National, regional, and worldwide estimates of stillbirth rates in 2015, with trends from 2000: a systematic analysis.. Lancet Glob Health.

[53] Tikkanen M (2011). Placental abruption: epidemiology, risk factors and consequences.. Acta Obstet Gynecol Scand.

[54] Turpin CA, Sakyi SA, Owiredu WK, Ephraim RK, Anto EO (2015). Association between adverse pregnancy outcome and imbalance in angiogenic regulators and oxidative stress biomarkers in gestational hypertension and preeclampsia.. BMC Pregnancy Childbirth.

[55] Odame Anto E, Owiredu WKBA, Sakyi SA, Turpin CA, Ephraim RKD, Fondjo LA, Obirikorang C, Adua E, Acheampong E (2018). Adverse pregnancy outcomes and imbalance in angiogenic growth mediators and oxidative stress biomarkers is associated with advanced maternal age births: a prospective cohort study in Ghana.. PLoS One.

[56] Straughen JK, Misra DP, Helmkamp L, Misra VK (2017). Preterm delivery as a unique pathophysiologic state characterized by maternal soluble FMS-Like tyrosine kinase 1 and uterine artery resistance during pregnancy: a longitudinal Cohort Study.. Reprod Sci.

[57] Shinohara S, Uchida Y, Kasai M, Sunami R (2017). Association between the high soluble fms-like tyrosine kinase-1 to placental growth factor ratio and adverse outcomes in asymptomatic women with early-onset fetal growth restriction.. Hypertens Pregnancy.

[58] Stepan H, Unversucht A, Wessel N, Faber R (2007). Predictive value of maternal angiogenic factors in second trimester pregnancies with abnormal uterine perfusion.. Hypertension.

[59] Valiño N, Giunta G, Gallo DM, Akolekar R, Nicolaides KH (2016). Biophysical and biochemical markers at 35-37 weeks’ gestation in the prediction of adverse perinatal outcome.. Ultrasound Obstet Gynecol.

[60] Stubert J, Ullmann S, Bolz M, Külz T, Dieterich M, Richter DU, Reimer T (2014). Prediction of preeclampsia and induced delivery at <34 weeks gestation by sFLT-1 and PlGF in patients with abnormal midtrimester uterine doppler velocimetry: a prospective cohort analysis.. BMC Pregnancy Childbirth.

[61] Spencer R, Ambler G, Weissbach T, Ginsberg Y, David A (2017). Prediction of perinatal mortality for the mid-trimester intrauterine growth restricted fetus using ultrasound and angiogenic markers.. Annual Meeting of the Blair Bell Research Society.

[62] Zeisler H, Llurba E, Chantraine F, Vatish M, Staff AC, Sennström M, Olovsson M, Brennecke SP, Stepan H, Allegranza D (2016). Soluble fms-Like tyrosine kinase-1-to-placental growth factor ratio and time to delivery in women with suspected preeclampsia.. Obstet Gynecol.

[63] Hoffmann J, Ossada V, Weber M, Stepan H (2017). An intermediate sFlt-1/PlGF ratio indicates an increased risk for adverse pregnancy outcome.. Pregnancy Hypertens.

[64] Zeisler H, Llurba E, Chantraine FJ, Vatish M, Staff AC, Sennström M, Olovsson M, Brennecke SP, Stepan H, Allegranza D (2019). Soluble fms-like tyrosine kinase-1 to placental growth factor ratio: ruling out pre-eclampsia for up to 4 weeks and value of retesting.. Ultrasound Obstet Gynecol.

[65] Álvarez-Fernández I, Prieto B, Rodríguez V, Ruano Y, Escudero AI, Álvarez FV (2016). N-terminal pro B-type natriuretic peptide and angiogenic biomarkers in the prognosis of adverse outcomes in women with suspected preeclampsia.. Clin Chim Acta.

[66] Sherrell H, Dunn L, Clifton V, Kumar S (2018). Systematic review of maternal placental growth factor levels in late pregnancy as a predictor of adverse intrapartum and perinatal outcomes.. Eur J Obstet Gynecol Reprod Biol.

[67] Rana S, Lemoine E, Granger JP, Karumanchi SA (2019). Preeclampsia: pathophysiology, challenges, and perspectives.. Circ Res.

[68] Rana S, Powe CE, Salahuddin S, Verlohren S, Perschel FH, Levine RJ, Lim KH, Wenger JB, Thadhani R, Karumanchi SA (2012). Angiogenic factors and the risk of adverse outcomes in women with suspected preeclampsia.. Circulation.

[69] Staff AC, Benton SJ, von Dadelszen P, Roberts JM, Taylor RN, Powers RW, Charnock-Jones DS, Redman CW (2013). Redefining preeclampsia using placenta-derived biomarkers.. Hypertension.

[70] Cerdeira AS, Agrawal S, Staff AC, Redman CW, Vatish M (2018). Angiogenic factors: potential to change clinical practice in pre-eclampsia?. BJOG.

[71] Verlohren S, Herraiz I, Lapaire O, Schlembach D, Zeisler H, Calda P, Sabria J, Markfeld-Erol F, Galindo A, Schoofs K (2014). New gestational phase-specific cutoff values for the use of the soluble fms-like tyrosine kinase-1/placental growth factor ratio as a diagnostic test for preeclampsia.. Hypertension.

[72] Stepan H, Hund M, Gencay M, Denk B, Dinkel C, Kaminski WE, Wieloch P, Semus B, Meloth T, Dröge LA (2016). A comparison of the diagnostic utility of the sFlt-1/PlGF ratio versus PlGF alone for the detection of preeclampsia/HELLP syndrome.. Hypertens Pregnancy.

