# Supplementary material for: Combining Biomarkers to Predict Pregnancy Complications and Redefine Preeclampsia: The Angiogenic-Placental Syndrome
Source: Hypertension. 2020 Feb 17;75(4):918–26. doi: 10.1161/HYPERTENSIONAHA.119.13763 (PMC7098437; doi:10.1161/HYPERTENSIONAHA.119.13763)
Supplement: Supplementary file 1 [file hyp-75-0918-s001.docx]

**Online Supplement**

## Combining Biomarkers to Predict Pregnancy Complications and Redefine Preeclampsia: the Angiogenic-Placental Syndrome

Holger Stepan, Martin Hund, Theresa Andraczek

From Leipzig University, Leipzig, Germany (H.S., T.A.); and Roche Diagnostics International Ltd, Rotkreuz, Switzerland (M.H.)

**Corresponding author:** Dr Holger Stepan, University of Leipzig, Liebigstrasse 20a, 04103, Leipzig, Germany, E-mail: [holger.stepan@medizin.uni-leipzig.de](mailto:holger.stepan@medizin.uni-leipzig.de)

**Supplementary References**

1. Gaccioli F, Sovio U, Cook E, Hund M, Charnock-Jones DS, Smith GCS. Screening for fetal growth restriction using ultrasound and the sFlt1/PlGF ratio in nulliparous women: A prospective cohort study. Lancet Child Adolesc Health. 2018;2:569-581

**Table S1. Ultrasonic and Biochemical Screening Test Diagnostic Effectiveness at 36 Weeks’ Gestation for Subsequent Delivery of a Small-for-Gestational-Age Infant Associated with Maternal Preeclampsia or Perinatal Morbidity/Mortality (n=3747)^1^**

| Outcome | Incidence, n (%) | True Positive / False Positive | True Negative / False Negative | Positive Likelihood Ratio   (95% CI) | Negative Likelihood Ratio (95% CI) | Sensitivity (95% CI) | Specificity (95% CI) | Positive Predictive Value  (95% CI) | Negative Predictive Value  (95% CI) |
| --- | --- | --- | --- | --- | --- | --- | --- | --- | --- |
| Ultrasonic estimated fetal weight below the 10th percentile | 521 (14) | 39/482 | 3207/19 | 5.1  (4.2–6.3) | 0.38  (0.26–0.54) | 67.2%  (53.8–78.3) | 86.9%  (85.8–88.0) | 7.5%  (5.5–10.1) | 99.4%  (99.1–99.6) |
| sFlt1/PlGF ratio >38 | 563 (15) | 31/532 | 3157/27 | 3.7  (2.9–4.8) | 0.54  (0.41–0.72) | 53.4%  (40.3–66.1) | 85.6%  (84.4–86.7) | 5.5%  (3.9–7.7) | 99.2%  (98.8–99.4) |
| Ultrasonic estimated fetal weight below 10th percentile and sFlt1/PlGF ratio >38 | 102 (3) | 22/80 | 3609/36 | 17.5  (11.8–25.9) | 0.63  (0.52–0.78) | 37.9%  (26.1–51.4) | 97.8%  (97.3–98.3) | 21.6%  (14.5–30.8) | 99.0%  (98.6–99.3) |
| Ultrasonic estimated fetal weight below 10th percentile and lowest decile of abdominal circumference growth velocity | NR | 18/143 | 3532/40 | 8.0  (5.3–12.1) | 0.72  (0.60–0.85) | 31.0%  (20.2–44.4) | 96.1%  (95.4–96.7) | 11.2%  (7.1–17.1) | 98.9%  (98.5–99.2) |
| Delphi procedure definition of late fetal growth restriction | NR | 35/377 | 3257/22 | 5.9  (4.7–7.4) | 0.43  (0.31–0.60) | 61.4%  (47.9–73.4) | 89.6%  (88.6–90.6) | 8.5%  (6.2–11.6) | 99.3%  (99.0–99.6) |

CI indicates confidence interval; NR, not reported; PlGF, placental growth factor; and sFlt-1, soluble fms-like tyrosine kinase 1.

Reprinted from Gaccioli F, et al. Screening for fetal growth restriction using ultrasound and the sFlt1/PlGF ratio in nulliparous women: A prospective cohort study. *Lancet Child Adolesc Health*. 2018;2:569-581, copyright 2018, with permission from Elsevier.^1^
