# Supplementary material for: Combining Biomarkers to Predict Pregnancy Complications and Redefine Preeclampsia: The Angiogenic-Placental Syndrome
Source: Hypertension. 2020 Feb 17;75(4):918–26. doi: 10.1161/HYPERTENSIONAHA.119.13763 (PMC7098437; doi:10.1161/HYPERTENSIONAHA.119.13763)
Supplement: Supplementary file 4 [file hyp-75-0918-s004.pdf]

Chloe Fletcher

From: no-reply@copyright.com
Sent: 20 December 2019 15:17
To: Anitha Narayan
Subject: Thank you for your order with RightsLink / The American Physiological Society

american physiological society

Thank you for your order!

Dear Miss. Anitha Narayan,

Thank you for placing your order through Copyright Clearance Center's RightsLink® service.

Order Summary

Licensee:	Anitha Narayan
Order Date:	Dec 20, 2019
Order Number:	4733100324737
Publication:	Physiology
Title:	Preeclampsia: The Role of Angiogenic Factors in Its Pathogenesis
Type of Use:	Journal/Magazine
Order Ref:	1109475
Order Total:	57.59 GBP

View or print complete details of your order and the publisher's terms and conditions.

Sincerely,

Copyright Clearance Center

Tel: +1-855-239-3415 / +1-978-646-2777
customercare@copyright.com
https://myaccount.copyright.com

Copyright Clearance Center

RightsLink®

This message (including attachments) is confidential, unless marked otherwise. It is intended for the addressee(s) only. If you are not an intended recipient, please delete it without further distribution and reply to the sender that you have received the message in error.
