# Supplementary material for: Combining Biomarkers to Predict Pregnancy Complications and Redefine Preeclampsia: The Angiogenic-Placental Syndrome
Source: Hypertension. 2020 Feb 17;75(4):918–26. doi: 10.1161/HYPERTENSIONAHA.119.13763 (PMC7098437; doi:10.1161/HYPERTENSIONAHA.119.13763)
Supplement: Supplementary file 5 [file hyp-75-0918-s005.pdf]

Chloe Fletcher

From: Chloe Fletcher
Sent: 22 January 2020 10:55
To: Chloe Fletcher
Subject: FW: RESPONSE REQUIRED for your request to Wolters Kluwer Health, Inc.

Categories: To Me

From: no-reply@copyright.com <no-reply@copyright.com>
Sent: 21 January 2020 15:02
To: Anitha Narayan <Anitha.Narayan@gcc-global.com>
Subject: RESPONSE REQUIRED for your request to Wolters Kluwer Health, Inc.

Wolters Kluwer

Accept your approved request

Dear Miss. Anitha Narayan,

Wolters Kluwer Health, Inc. has approved your recent request described below. Before you can use this content, **you must accept** the license fee and terms set by the publisher.

Use this link to accept (or decline) the publisher's fee and terms for this order.

Order Summary

Licensee:	Anitha Narayan
Order Date:	Jan 14, 2020
Order Number:	501539684
Publication:	Obstetrics & Gynecology
Title:	Soluble fms-Like Tyrosine Kinase-1-to-Placental Growth Factor Ratio and Time to Delivery in Women With Suspected Preeclampsia
Type of Use:	Journal/Magazine
Order Ref:	1109475
Order Total:	384.84 GBP

View or print complete details of your request.

Sincerely,

Copyright Clearance Center

Tel: +1-855-239-3415 / +1-978-646-2777
customercare@copyright.com
https://myaccount.copyright.com

Copyright Clearance Center

RightsLink®

This message (including attachments) is confidential, unless marked otherwise. It is intended for the addressee(s) only. If you are not an intended recipient, please delete it without further distribution and reply to the sender that you have received the message in error.
