# Supplementary material for: Combining Biomarkers to Predict Pregnancy Complications and Redefine Preeclampsia: The Angiogenic-Placental Syndrome
Source: Hypertension. 2020 Feb 17;75(4):918–26. doi: 10.1161/HYPERTENSIONAHA.119.13763 (PMC7098437; doi:10.1161/HYPERTENSIONAHA.119.13763)
Supplement: Supplementary file 6 [file hyp-75-0918-s006.pdf]

The American College of Obstetricians and Gynecologists
WOMEN'S HEALTH CARE PHYSICIANS

Invoice No. 121819

ACOG Products and Publications Department
REPRINT PERMISSION

December 18, 2019

Anitha Narayan
Senior Editorial Assistant
Gardiner-Caldwell Communications

Dear Ms Narayan:

Permission is granted to reprint Box 2, "Diagnostic Criteria for Preeclampsia" from Practice Bulletin no. 202 *Gestational Hypertension and Preeclampsia* for use in the article "Combining Biomarkers to Predict Pregnancy Complications and Redefine Preeclampsia: the Angiogenic-Placental Syndrome," to be published in the print and electronic versions of *Hypertension*. The following citation must be used:

Reprinted with permission from Gestational hypertension and preeclampsia. ACOG Practice Bulletin No. 202. American College of Obstetricians and Gynecologists. Obstet Gynecol 2019;133:e1-25.

Permission to reproduce the aforementioned content selection is granted based on the following conditions:

1. Full credit to the source is shown as indicated above.
2. The information must be reproduced **without revision or modification**.
3. Permission is given for **one-time** print and electronic English use only for the purpose requested and does not extend to subsequent editions, foreign language translations, or derivative works.
4. Permission is contingent on payment of a fee of $200 (print and electronic rights).

Sincerely,

Rachel Weydert

Rachel Weydert
ACOG Permissions

This letter serves as your invoice. Please include a copy of it with your payment made payable to
American College of Obstetricians and Gynecologists
Attention: Products and Publications Department
Invoice reference no. 121819 $200 due on receipt of letter

409 12th Street, SW, Washington, DC 20024
Mailing address: PO Box 96920, Washington, DC 20090-6920
(202) 638-5577 • www.acog.org
