# Supplementary material for: Combining Biomarkers to Predict Pregnancy Complications and Redefine Preeclampsia: The Angiogenic-Placental Syndrome
Source: Hypertension. 2020 Feb 17;75(4):918–26. doi: 10.1161/HYPERTENSIONAHA.119.13763 (PMC7098437; doi:10.1161/HYPERTENSIONAHA.119.13763)
Supplement: Supplementary file 7 [file hyp-75-0918-s007.pdf]

Chloe Fletcher

**From:**	Chloe Fletcher
**Sent:**	16 January 2020 15:17
**To:**	Chloe Fletcher
**Subject:**	FW: Thank you for your order with RightsLink / Massachusetts Medical Society
**Categories:**	To Me

From: no-reply@copyright.com <no-reply@copyright.com>
Sent: 16 January 2020 12:20
To: Anitha Narayan <Anitha.Narayan@gcc-global.com>
Subject: Thank you for your order with RightsLink / Massachusetts Medical Society

NEJM

Thank you for your order!

Dear Miss. Anitha Narayan,

Thank you for placing your order through Copyright Clearance Center's RightsLink® service.

Order Summary

Licensee:	Anitha Narayan
Order Date:	Jan 16, 2020
Order Number:	4750740929567
Publication:	The New England Journal of Medicine
Title:	Predictive Value of the sFlt-1:PlGF Ratio in Women with Suspected Preeclampsia
Type of Use:	Journal/Magazine
Order Ref:	1109475
Order Total:	1,469.30 GBP

(Original Order Number: 501539682)

View or print complete details of your order and the publisher's terms and conditions.

Sincerely,

Copyright Clearance Center

Tel: +1-855-239-3415 / +1-978-646-2777
customercare@copyright.com
https://myaccount.copyright.com

Copyright Clearance Center RightsLink®

This message (including attachments) is confidential, unless marked otherwise. It is intended for the addressee(s) only. If you are not an intended recipient, please delete it without further distribution and reply to the sender that you have received the message in error.
