# Supplementary material for: Combining Biomarkers to Predict Pregnancy Complications and Redefine Preeclampsia: The Angiogenic-Placental Syndrome
Source: Hypertension. 2020 Feb 17;75(4):918–26. doi: 10.1161/HYPERTENSIONAHA.119.13763 (PMC7098437; doi:10.1161/HYPERTENSIONAHA.119.13763)
Supplement: Supplementary file 8 [file hyp-75-0918-s008.pdf]

Chloe Fletcher

From: Lingayath, Roopa (ELS-CHN) <r.lingayath@elsevier.com>
Sent: 20 December 2019 10:22
To: Anitha Narayan
Cc: Chloe Fletcher; Lucy Carrier; Adam Davies
Subject: RE: Permission: Table S1 from Gaccioli et al. 2018

ELSEVIER

Dear Anitha Narayan

We hereby grant you permission to reproduce the material detailed below in **print and electronic format** at no charge subject to the following conditions:

RE: Table 3 from Gaccioli et al. Screening for fetal growth restriction using ultrasound and the sFLT1/PlGF ratio in nulliparous women: a prospective cohort study. 2018

Proposed use: To be used in a manuscript recently accepted by Hypertension, titled Combining Biomarkers to Predict Pregnancy Complications and Redefine Preeclampsia: the Angiogenic-Placental Syndrome.

1. If any part of the material to be used (for example, figures) has appeared in our publication with credit or acknowledgement to another source, permission must also be sought from that source. If such permission is not obtained then that material may not be included in your publication/copies.

2. Suitable acknowledgement to the source must be made, either as a footnote or in a reference list at the end of your publication as follows:

   "This article was published in Publication title, Vol number, Author(s), Title of article, Page Nos, Copyright Elsevier (or appropriate Society name) (Year)."

3. This permission is granted for non-exclusive world rights in all languages.

4. Reproduction of this material is granted for the purpose for which permission is hereby given, and includes use in any future editions.

Kind Regards
Roopa

Thanks & Regards,
Roopa Lingayath
Sr Copyrights Coordinator – Copyrights Team
ELSEVIER | Health Content Operations
International Tech Park | Crest – 5th Floor | CSIR Road | Taramani | Chennai 600 113 | India
Tel: +91 44 3378 4167 | Fax: +91 44 4299 4568
E-mail: r.lingayath@elsevier.com | url: www.elsevier.com

From: Anitha Narayan <Anitha.Narayan@gcc-global.com>
Sent: Thursday, December 19, 2019 6:15 PM
To: Lingayath, Roopa (ELS-CHN) <r.lingayath@elsevier.com>
Cc: Chloe Fletcher <Chloe.Fletcher@gcc-global.com>; Lucy Carrier <Lucy.Carrier@gcc-global.com>; Adam Davies <Adam.Davies@gcc-global.com>
Subject: RE: Permission: Table S1 from Gaccioli et al. 2018

***** External email: use caution *****

RE: Table 3 from Gaccioli et al. Screening for fetal growth restriction using ultrasound and the sFLT1/PlGF ratio in nulliparous women: a prospective cohort study. 2018

Dear Roopa,

Thank you so much for granting permission for us to reuse the table outlined below. Please accept my apologies - the table we would like permission to reuse is **Table 3** (not Table S1 as stated below). Please could you let me know if we could update the permission below to include Table 3 instead?

With best regards and thanks,
Anitha

Anitha Narayan
Senior Editorial Assistant, Gardiner-Caldwell Communications

T +44 1625 664047
E Anitha.Narayan@gcc-global.com

Ashfield Healthcare Communications, part of UDG Healthcare plc
---
www.gcc-global.com
Commercial / Clinical / Healthcare Communications / Insight & Performance / Market Access / Medical Information / Meetings & Events / Pharmacovigilance

Ashfield

From: Lingayath, Roopa (ELS-CHN) <r.lingayath@elsevier.com>
Sent: 19 December 2019 08:30
To: Anitha Narayan <Anitha.Narayan@gcc-global.com>
Cc: Chloe Fletcher <Chloe.Fletcher@gcc-global.com>; Lucy Carrier <Lucy.Carrier@gcc-global.com>
Subject: RE: Permission: Table S1 from Gaccioli et al. 2018

ELSEVIER

Dear Anitha Narayan

We hereby grant you permission to reproduce the material detailed below in **print and electronic format** at no charge subject to the following conditions:

RE: Table S1 from Gaccioli et al. Screening for fetal growth restriction using ultrasound and the sFLT1/PlGF ratio in nulliparous women: a prospective cohort study. 2018

Proposed use: To be used in a manuscript recently accepted by Hypertension, titled Combining Biomarkers to Predict Pregnancy Complications and Redefine Preeclampsia: the Angiogenic-Placental Syndrome.

1. If any part of the material to be used (for example, figures) has appeared in our publication with credit or acknowledgement to another source, permission must also be sought from that source. If such permission is not obtained then that material may not be included in your publication/copies.

2. Suitable acknowledgement to the source must be made, either as a footnote or in a reference list at the end of your publication as follows:

   "This article was published in Publication title, Vol number, Author(s), Title of article, Page Nos, Copyright Elsevier (or appropriate Society name) (Year)."

3. This permission is granted for non-exclusive world rights in all languages.

4. Reproduction of this material is granted for the purpose for which permission is hereby given, and includes use in any future editions.

Kind Regards
Roopa

Thanks & Regards,
Roopa Lingayath
Sr Copyrights Coordinator – Copyrights Team
ELSEVIER | Health Content Operations
International Tech Park | Crest – 5th Floor | CSIR Road | Taramani | Chennai 600 113 | India
Tel: +91 44 3378 4167 | Fax: +91 44 4299 4568
E-mail: r.lingayath@elsevier.com | url: www.elsevier.com

From: Anitha Narayan <Anitha.Narayan@gcc-global.com>
Sent: Thursday, November 21, 2019 10:33 PM
To: Rights and Permissions (ELS) <Permissions@elsevier.com>
Cc: Chloe Fletcher <Chloe.Fletcher@gcc-global.com>; Lucy Carrier <Lucy.Carrier@gcc-global.com>
Subject: Permission: Table S1 from Gaccioli et al. 2018

***** External email: use caution *****

Table S1 from *Gaccioli et al. Screening for fetal growth restriction using ultrasound and the sFLT1/PlGF ratio in nulliparous women: a prospective cohort study. 2018*
https://www.thelancet.com/journals/lanchi/article/PIIS2352-4642(18)30129-9/fulltext#%20
Dear Sir/Madam,
I am writing on behalf of Prof. Stepan to request permission to include Table S1 from the above manuscript in a manuscript recently accepted by *Hypertension*, titled Combining Biomarkers to Predict Pregnancy Complications and Redefine Preeclampsia: the Angiogenic-Placental Syndrome.
Please do let me know if you need more information. We would be very grateful if you could provide a quote as soon as possible.
With best regards and thanks,
Anitha

Anitha Narayan
Senior Editorial Assistant, Gardiner-Caldwell Communications

T +44 1625 664047
E Anitha.Narayan@gcc-global.com

Ashfield Healthcare Communications, part of UDG Healthcare plc
---
www.gcc-global.com
Commercial / Clinical / Healthcare Communications / Insight & Performance / Market Access / Medical Information / Meetings & Events / Pharmacovigilance

Ashfield

Ashfield Healthcare Communications Group Limited is a limited company incorporated and registered in England and Wales with registered company number 01887613 and VAT registered number GB 179-3512-83. Our registered office is at Ashfield House, Resolution Road, Ashby de la Zouch, Leicestershire, LE65 1HW, UK.

This message is intended solely for the addressee and may contain confidential information. If you have received this message in error, please send it back to us, and immediately and permanently delete it. Do not use, copy or disclose the information contained in this message or in any attachment. For information about how we process data and monitor communications please see our Privacy Policy on our website at http://www.ashfieldhealthcare.com .
